# Biomarkers for PTSD at the Interface of the Endocannabinoid and Neurosteroid Axis

**DOI:** 10.3389/fnins.2018.00482

**Published:** 2018-08-06

**Authors:** Graziano Pinna

**Affiliations:** The Psychiatric Institute, Department of Psychiatry, College of Medicine, University of Illinois at Chicago, Chicago, IL, United States

**Keywords:** allopregnanolone, biomarkers, N-palmitoylethanolamine, PPAR-α, endocannabinoids, 5α-reductase, contextual fear responses, PTSD

## Introduction

In the search for reliable and, possibly, specific biomarkers for neuropsychiatric disorders, growing evidence has demonstrated that biosynthesis of neuroactive steroids and the endocannabinoid system are involved in the neuropathology of post-traumatic stress disorder (PTSD) and major depressive disorder (Uzunova et al., 1998; Rasmusson et al., 2006; reviewed in Locci and Pinna, 2017a). Although, undisputable progress has been made to assess validity of biomarkers for psychiatric disorders, the topic still remains underdeveloped as compared to other fields of neuroscience (Fernandes et al., 2017). The diagnosis of psychiatric disorders still relies on subjective measures centered on the DSM-5 criteria which have several shortcomings (Brewin et al., 2017). Psychiatric conditions are poorly understood and there is a wide heterogeneity in how illness manifests in several individuals. Furthermore, self-assessment of one's own feelings can be biased, ill-defined, and difficult, making psychological diagnoses unreliable and may lead to treatment inefficacy. Biomarkers discovery would significantly improve treatment matching. Thus, searching for potential biomarkers to guide precision medicine in the treatment of PTSD, and to increase the success of clinical trials and prompt the development of novel and specific treatments, is required. To aid this search, more sophisticated methodological tools and validated animal models has also become essential to reliably correlate behavioral changes with neurochemical alterations (reviewed in Ngounou Wetie et al., 2013). The overlap of symptoms and the comorbidity with other psychiatric disorders such as major depressive disorder, anxiety spectrum disorders, and even suicidal ideation (Franklin et al., 2017), suggest a bio-signature for PTSD should include the relation of numerous biomarkers rather than having only a few (Locci and Pinna, 2017a). A refined approach to more specifically “bio-define” PTSD can be to establish a *biomarker axis* or in other words, to assess the relation of various biomarkers, which fluctuate in concert and correlate uniquely with PTSD behavioral modifications. Insofar, a *biomarker axis* may provide a higher accuracy in the diagnosis of the disorder with benefits for prediction in PTSD treatment response and relapse (Locci et al., 2018; Pinna and Izumi, 2018). As a matter of fact, the “gold standard” treatment for PTSD and depression, the selective serotonin reuptake inhibitors (SSRIs), improve only half of the treatment-seeking patients and they are associated with severe side-effects (Golden et al., 2002; Rush et al., 2006; Kemp et al., 2008; reviewed in Bernardy and Friedman, 2017). This also suggests these psychiatric disorders are complex, multifaceted diseases arising from multiple and diverse neurobiological backgrounds and therefore, symptoms may not always recapitulate to a serotonergic deficit and administering an SSRI may not always improve symptoms. Unveiling reliable biomarkers is also a necessity for patient stratification in treatment selection as well as for drug development through clinical trials. The development of state-of-the-art technologies and methodological rigor are essential to allow for the discovery of more reliable biomarkers in psychiatry. Employing the gas chromatography-mass spectrometry (GC-MS) to achieve this goal is highly innovative and provides reliable information based on a powerful technology with high sensitivity and unsurpassed structure selectivity (Uzunov et al., 1996; Pinna et al., 2000). Hence, by applying the GC-MS measurements of neuroactive steroids in serum, plasma, CSF and post-mortem brain, in the past decade, we have shed light in the fundamental role of neuroactive steroids in patients with neuropsychiatric disorders (Rasmusson et al., 2006, 2017; Agis-Balboa et al., 2014; Pineles et al., 2018; reviewed in Locci and Pinna, 2017a).

The biosynthesis of allopregnanolone, a positive allosteric modulator of GABA's action at GABA_A_ receptors has been found deficient in a number of neuropsychopathologies, including epilepsy (e.g., PHDH19), major depression, PTSD, perceived social isolation, post-partum depression, premenstrual syndrome, and anorexia nervosa or obesity complicated by anxiety and depression symptoms in women (Romeo et al., 1998; Uzunova et al., 1998; Rasmusson et al., 2006, 2018; Nemeroff, 2008; Lovick, 2013; Trivisano et al., 2017; Dichtel et al., 2018; Pineles et al., 2018). Therapeutic measures aimed at reinstating normal allopregnanolone levels in deficient-patients correlates with improved symptoms (Kanes et al., 2017). The question arises as to whether allopregnanolone biosynthesis *per se* is a reliable biomarker to predict, diagnose and instruct treatment selection of patients or whether its relation with neurotransmitter systems (GABA_A_ and NMDA receptors), stimulation of neurotropic factors (e.g., BDNF), and/or crosstalk with the endocannabinoid system (e.g., PPAR-α) may provide a valuable *biomarker axis* with a higher disorder-selectivity. This analysis includes both neurosteroids that are positive allosteric modulators of GABA_A_ receptors (Pinna et al., 2000; Belelli and Lambert, 2005), such as allopregnanolone and pregnanolone and their sulfated forms that are inhibitors of NMDA-mediated tonic neurotransmission, which results in neuroprotection (Vyklicky et al., 2016).

**Figure 1:**
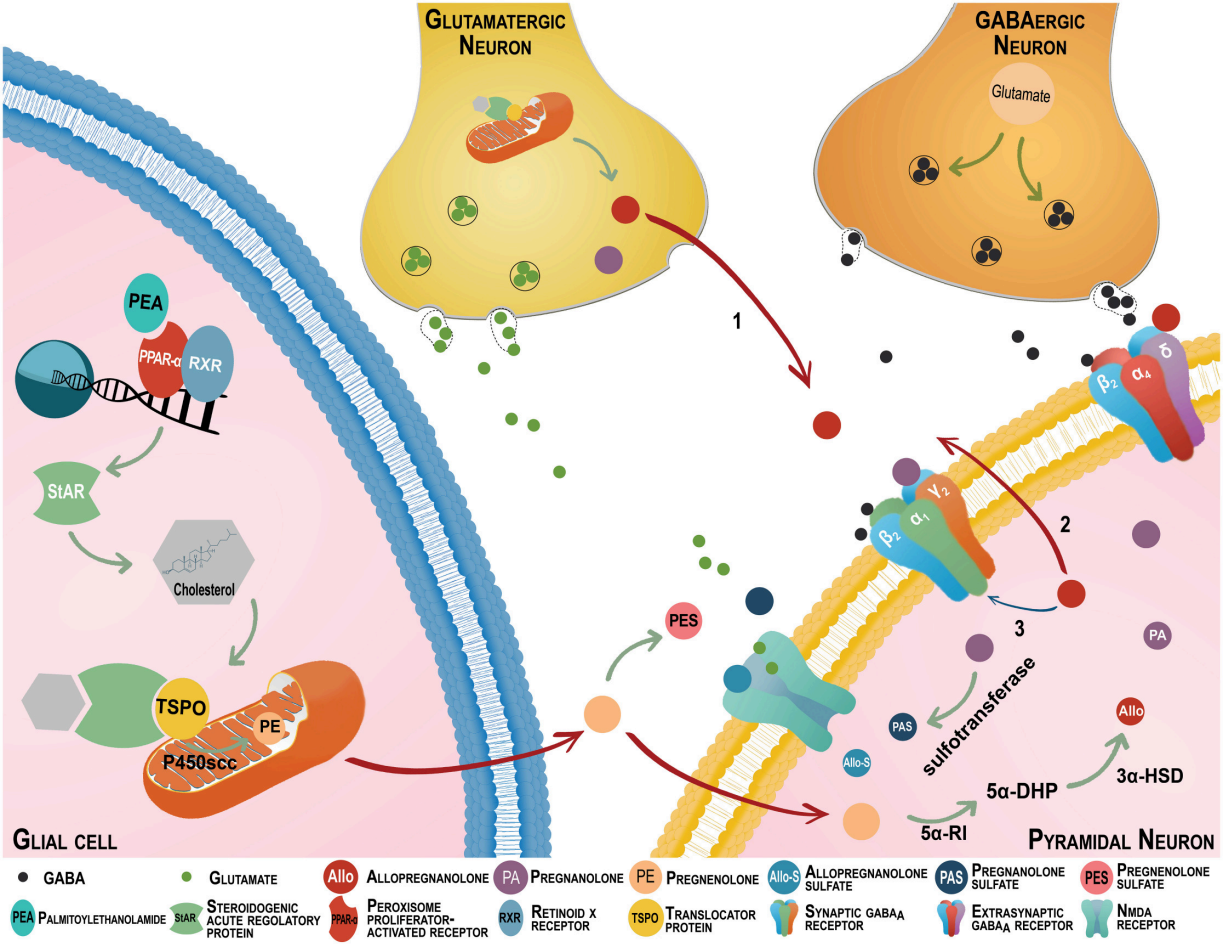
The endocannabinoid and neurosteroid systems cross-talk regulates emotional behavior. The neurosteroids, allopregnanolone (Allo) and its equipotent isomer pregnanolone (PA) are primarily synthesized in glutamatergic corticolimbic neurons and upon secretion; they may act at GABA_A_ receptors located on cell bodies or dendrites of distal pyramidal neurons (Arrow 1). They may also act at GABA_A_ receptors located on glutamatergic neurons' dendrites or cell bodies by an autocrine mechanism (Arrow 2), or may access and act at the intracellular sites of GABA_A_ receptors located in glutamatergic neurons that produced allopregnanolone itself (Arrow 3) (Agís-Balboa et al., 2006, 2007; Pinna et al., 2008). Allopregnanolone plays a central neuromodulatory role in facilitating the action of GABA at GABA_A_ receptors (a primary target of anxiolytics) and in the fine-tuning of the receptor for agonists and GABAmimetic agents (Pinna et al., 2000). The finding that allopregnanolone facilitates the efficacy of GABA_A_ receptor allosteric modulators substantiates its endogenous physiological relevance (Pinna et al., 2000, 2008; Guidotti et al., 2001). Importantly, GABA_A_ receptors composed by α,β,γ subunits are the most common configuration in the synaptic membranes and they are responsible for the inhibitory *phasic* currents. These receptors are benzodiazepine-sensitive but show lower sensitivity to GABA and allopregnanolone (Nusser and Mody, 2002). The GABA_A_ receptors including α,β,δ subtypes are mostly extrasynaptic and mediate inhibitory *tonic* currents. Of note, they are not sensitive to benzodiazepines and show low efficacy for GABA, however, allopregnanolone increase their efficacy (Stell et al., 2003; Shu et al., 2012). The efficacy of GABAergic neurosteroids is greatly enhanced for this receptor combination (Brown et al., 2002; Nusser and Mody, 2002; Wohlfarth et al., 2002). Remarkably, protracted stress favors a GABA_A_ receptor composition with high sensitivity for allopregnanolone and its analogs (Locci and Pinna, 2017a). Following the action of sulphotransferase, allopregnanolone, and pregnanolone can be transformed into allopregnanolone sulfate (Allo-S) and pregnanolone sulfate (PAS). These sulfated steroids can be measured by gas chromatography-mass spectrometry in serum, CSF, and brain of patients or rodents in concentrations consistent with a physiological role in modulating neurotransmitter systems (Smith et al., 2014; Locci and Pinna, 2017b). Recently, pregnanolone sulfate has been shown to inhibit NMDA receptors. Pregnanolone sulfate can accumulate in plasma membranes and may accesses binding sites that are located at NMDA receptors (Borovska et al., 2012). Importantly, pregnanolone sulfate, and probably allopregnanolone sulfate, is highly potent at inhibiting tonic rather than synaptically mediated NMDA receptor neurotransmissions. While synaptic NMDA receptors play a pivotal role in synaptic plasticity, learning and memory, as well as in synaptogenesis, tonic-mediated NMDA receptor neurotransmission is mostly involved with excitotoxicity. Thus, the effects of pregnanolone sulfate negative modulation of tonic-mediated NMDA receptor neurotransmission have relevance for neuroprotection (Vyklicky et al., 2016). By this mechanism, these allopregnanolone and pregnanolone sulfated derivatives may play a role in the regulation of cognitive processes and of emotional behavior (reviewed in Locci and Pinna, 2017a). There is growing evidence that the intracellular peroxisome proliferator-activated receptor (PPAR-α) is also a cannabinoid target (depicted on the bottom right). PPAR-α heterodimerize with the retinoid X receptor (RXR) and binds to the consensus regions on the target gene promoters and initiates transcription (Neumeister, 2013). Given that endoannabinoids activate PPAR-α (Marsicano et al., 2002; Pistis and Melis, 2010), the activation of these nuclear receptors represents a novel mechanism by which cannabinoids may modulate behavior. The endocannabinoid congener, N-palmitoylethanolamine (PEA) is a PPAR-α endogenous agonist, which is decreased in PTSD patients (Wilker et al., 2016). Recent preclinical findings showed that supplementing PEA in rodent PTSD models improves emotional behavior by enhancing allopregnanolone biosynthesis in corticolimbic glutamatergic neurons. This effect is mimicked by PPAR-α agonists and prevented by allopregnanolone biosynthetic enzyme blockers and by deletion of the PPAR-α gene (Locci and Pinna, 2017b). Thus, anxiolytic, anti-aggressive and anti-fear effects of PEA and synthetic PPAR-α agonists may relate to an induction of corticolimbic allopregnanolone's biosynthetic enzymes. This may result in potentiation of GABA_A_ receptor and, possibly, in an inhibition of tonic-mediated NMDA signal transduction associated with improved behavioral dysfunction. Stress effects on PEA levels and probably expression of PPAR-α may result in the downregulation of allopregnanolone's biosynthetic enzyme expression and allopregnanolone levels. The interface of the endocannabinoid and neurosteroid systems may provide an important *biomarker axis* to selectively predict, diagnose, and establish the best individualized treatment selection for PTSD patients.

The novel discovery that the endocannabinoid system regulates the biosynthesis of neurosteroids, including allopregnanolone has recently opened the field for assessing valuable PTSD biomarkers at the interface of these neuronal systems. In recent years, cannabinoid-based agents have become an integral part of drug discovery for PTSD treatment (Ruehle et al., 2012; Neumeister et al., 2014). The impact of the endocannabinoid system is under-scored by the density of receptors in glutamatergic neurons of emotion-relevant areas, including the the amygdaloid complex, the hippocampus and the frontal cortex (Katona, 2009). Synthetic cannabinoid receptor antagonists or knockouts enhance fear acquisition and impair fear extinction, a core feature of PTSD (Reich et al., 2008; Papini et al., 2015). In addition to the well assessed role of the endocannabinoid, anandamide (AEA) or 2-arachidonoyl-glycerol (2-AG) both in neuropsychiatric disorders and animal models of stress (Chhatwal et al., 2005; Umathe et al., 2011; Dubreucq et al., 2012), compelling evidence indicates stimulation of the intracellular endocannabinoid target, peroxisome-proliferator activated receptor (PPAR)-α by its endogenous neuromodulator, N-palmitoylethanolamine (PEA) engages the biosynthesis of neurosteroids to modulate emotional behavior (Locci and Pinna, 2017b; Locci et al., 2018) (please see Figure 1 for a graphic representation).

This unforeseen behavioral and neurosteroidogenic function of PPAR-α, formally known to regulate pathophysiological functions, including inflammation and oxidative stress, opens the field for potential novel biomarkers for PTSD.

**Figure 2:**
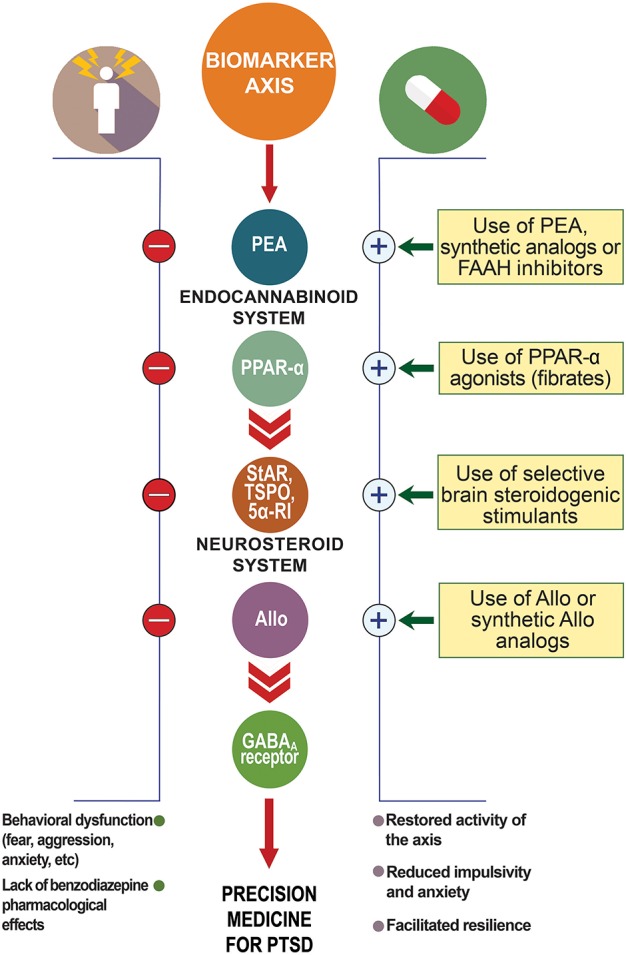
Biomarker axis at the interface of the endocannabinoid and neurosteroid systems. In animal models of PTSD, protracted stress results in the downregulation of allopregnanolone biosynthetic enzymes (e.g., 5α-reductase type I, 5α-RI) and allopregnanolone concentrations in corticolimbic glutamatergic neurons of the frontal cortex, hippocampus, and basolateral amygdala. This allopregnanolone decrease correlates with behavioral dysfunction, such as increased aggression, enhanced contextual fear responses and anxiety-like behavior (Pinna et al., 2003; Pibiri et al., 2008). Supplying allopregnanolone or stimulating its biosynthesis decreases anxiety-like behavior, aggression and fear responses (Pinna, 2013; Pinna and Rasmusson, 2014). Stress may also result in changes in GABA_A_ receptor subunit expression (Pinna et al., 2006; reviewed in Locci and Pinna, 2017a) with increased α4, α5, and δ subunits and decreased α1, α2, and γ2, which result in down-regulated benzodiazepine binding sites and inefficacy of benzodiazepine pharmacological action (Pinna et al., 2006; Nin et al., 2011b). Protracted stress results in increased GABA_A_ receptor subunits, including α_4−5_,β,δ highly sensitivity for allopregnanolone (Locci and Pinna, 2017a). Both allopregnanolone biosynthesis downregulation and decreased benzodiazepine binding sites have been reported in PTSD patients (Rasmusson et al., 2006, 2018; Geuze et al., 2008). Thus, the combination of downregulation of allopregnanolone biosynthesis, changes in GABA_A_ receptor subunit expression, and lack of benzodiazepine pharmacological action are peculiar changes observed in PTSD that may provide a selective *biomarker axis* for this disorder. Stress may affect PEA levels and expression of PPAR-α, which in turn may downregulate allopregnanolone concentrations. Thus, the PPAR-α-allopregnanolone axis may provide further biomarker candidates to support selection of the best individualized precision medicine for PTSD. Allo, allopregnanolone; GABA, γ-aminobutyric acid; PEA, N-palmitoylethanolamine; PPAR-α, peroxisome-proliferator activated receptor-α; StAR, steroidogenic acute regulatory protein; TSPO, 18 kDa translocator protein.

This article will discuss whether new discoveries in the field support a biomarker role for allopregnanolone biosynthesis and the endocannabinoid system for stress-induced disorders with focus on PTSD. The strategy of assessing a *biomarker axis*, which indicates the relation of various inter-related neurobiological deficits for one disorder (Figure 2), may help for diagnosis accuracy and for designing successful individualized treatments.

## Neurosteroid action at GABA_A_ and NMDA receptors

Sulfated or unconjugated neuroactive steroids modulate ionotropic amino acid neurotransmitter receptors, including GABA_A_ and NMDA receptors. The GABA_A_ receptor offers two binding residues that express affinity for allopregnanolone and unconjugated congeners (e.g., pregnanolone) that act as potent positive allosteric modulators of the action of GABA at GABA_A_ receptors. One is located at the interface of the α/β subunits, and the other is within the cavity of α subunits (Hosie et al., 2006). The α,β,γ GABA_A_ receptor subtype is the most frequent synaptic configuration and is highly sensitive to benzodiazepines but shows lower sensitivity to GABA and neurosteroids (Nusser and Mody, 2002). The α,β,δ GABA_A_ receptor subtype expressed in the extrasynaptic region is benzodiazepine-insensitive, show low efficacy for GABA, but neurosteroids increase its agonist efficacy (Stell et al., 2003; Shu et al., 2012). This receptor combination shows high efficacy for neurosteroids (Brown et al., 2002; Nusser and Mody, 2002; Wohlfarth et al., 2002; Figure 1). Sulfated neurosteroids such as pregnenolone sulfate, dehydroepiandrosterone sulfate, pregnanolone sulfate, and allopregnanolone sulfate may function as endogenous neuromodulators by inhibiting GABA_A_ receptors, or depending on the receptor conformation and the sulfated neuroactive steroid examined, by activating or inhibiting NMDA-mediated neurotransmission (Park-Chung et al., 1999). Sulfation at C3 is essential to reverse the direction of modulation from positive to negative in GABA_A_ receptors. Steroid negative and positive modulators act through distinct sites, which implies that steroid negative and positive modulators can act independently or coordinately to modulate the flavor of GABAergic-mediated inhibitory neurotransmission (reviewed in Smith et al., 2014). While, micromolar concentrations of pregnenolone sulfate negatively modulate GABA_A_ receptors, pregnenolone sulfate can negatively or positively modulate NMDA receptors, depending on the receptor subunits expressed (Malayev et al., 2002; Smith et al., 2014). For instance, pregnenolone sulfate potentiates NMDA receptors that contain NR2A and NR2B subunits, but negatively modulates NR2C and NR2D-containing receptors (Malayev et al., 2002). Recent studies showed that pregnanolone sulfate has a potent inhibitory action at tonic rather than synaptically-activated NMDA receptors, which provides neuroprotection and possibly improves emotional behavior and cognition (Vyklicky et al., 2016). This feature is relevant for developing a novel class of steroid-based NMDA-inhibitors devoid of the psychotomimetic effects that characterize classical NMDA receptor inhibitors, including ketamine. While GABA_A_ receptor subunit expression during protracted stress has been previously investigated (discussed below), the role and action of sulfated pregnanolone, pregnenolone, allopregnanolone, and the expression of NMDA receptor subunits in PTSD patients and in rodent stress models, still warrants elucidation.

### The neurosteroid and endocannabinoid crosstalk

Intriguingly, studies conducted in cell cultures, brainstem and spinal cord showed the endocannabinoid, PEA binding at the ligand-activated nuclear receptor, PPAR-α stimulates allopregnanolone biosynthesis and potentiates pentobarbital-induced sedation (Sasso et al., 2010, 2012; Raso et al., 2011). These observations suggest that PPAR-α may play a role in the regulation of emotions by inducing neurosteroidogenesis in corticolimbic neurons following binding with its endogenous ligand, PEA, or synthetic agonists. Whereas the classic cannabinoid receptor type 1 (CB1) has been shown to regulate emotions and stress responses, PPAR-α's role on emotions remains poorly understood (Riebe and Wotjak, 2011; Häring et al., 2012). The relevance of the endocannabinoid system in behavior is highlighted by expression of CB1 and PPAR-α in glutamatergic neurons of emotion-relevant areas that have been identified by brain imaging to be critical in PTSD (amygdala, hippocampus, frontal cortex) (Moreno et al., 2004; Lo Verme et al., 2005; Shin et al., 2006; D'Agostino, 2007; D'Agostino et al., 2009; Katona, 2009; Petrosino and Di Marzo, 2017). Moreover, evidence suggests CB1 disruption leads to impaired fear extinction (Reich et al., 2008), depressive- and anxiety-like behavior, while agonists, like AEA, induce anxiolysis and improve fear responses (Hill and Patel, 2013). Current thought suggests that the effects of AEA at CB1 account for the majority of anti-fear effects of endocannabinoids (Marsicano et al., 2002; Viveros et al., 2005; Kamprath et al., 2006; Thiemann et al., 2008; Jacob et al., 2012), however this view seems no longer tenable (Pistis and Melis, 2010). In addition to these cell-surface cannabinoid receptors (O'Sullivan, 2007), there is growing evidence that PPAR-α's activation represents a novel mechanism by which cannabinoids modulate behavior. Stimulation of PPAR-α by PEA or synthetic PPAR-α agonists was recently shown to elevate corticolimbic allopregnanolone levels in hippocampus, amygdala, frontal cortex and in olfactory bulb, which correlated with improvement of PTSD-like behavior in socially isolated mice (Locci and Pinna, 2017a). PEA facilitates contextual fear extinction and fear extinction retention and induces anti-aggressive, anxiolytic, and antidepressant-like effects in socially isolated mice (Locci and Pinna, 2017b; Locci et al., 2017). PPAR-α synthetic agonists normalized allopregnanolone levels and improved behavior, whereas antagonism at PPAR-α, inhibition of allopregnanolone biosynthetic enzymes, or PPAR-α KO mice prevented both PEA-induced behavior and its neurosteroidogenic effects (Locci and Pinna, 2017b). While the role of PPAR-α in neuropsychiatric disorders is just emerging, studies in the field suggest serum PEA and oleoylethanolamide (OEA) concentrations increase after acute social stressor (Dlugos, 2012) and decrease following recovery (Hill et al., 2009a). Stress evokes fast induction of fatty acid amide hydrolase (FAAH), which reduces PEA and AEA levels (Patel et al., 2005; Hill et al., 2009b). In PTSD patients, symptoms are inversely correlated with reduced hair levels of PEA, OEA and stearoylethanolamide (SEA) in both males and females (Wilker et al., 2016). PEA adjunctive therapy to citalopram improves symptoms in depressed patients (Ghazizadeh-Hashemi, 2018). Furthermore, intense workouts increase PEA and OEA levels and improve depression and PTSD (Heyman, 2012). In rodents, exposure to predator stressors reduces cardiac PEA and OEA levels (Holman et al., 2014), but, antidepressant-like effects are induced by increasing PEA and OEA (Adamczyk et al., 2008; Umathe et al., 2011; Melis et al., 2013). Collectively, the crosstalk between the endocannabinoid system and neurosteroid biosynthesis during stress may unveil *biomarker axis* uniquely altered in specific stress-induced mood disorders.

## Biomarkers and treatment options for PTSD at the interface of the endocannabinoid and neurosteroid axis

Psychiatric disorders, such as PTSD, are not currently amenable to objective neurobiological determinations as is routine practice in the diagnosis and treatment of other medical conditions. This is most likely due to the general complexity and multifactorial origins of these disorders and the difficulty to establish a consistent bio-signature. While no biomarkers for PTSD have to date been firmly assessed with diagnostic validity, a consistent progress in the field has been done. Biomarker candidates for PTSD have been proposed but often they share overlaps with other psychiatric disorders with similar symptoms and that are currently treated with same drugs. Indeed, the first-choice pharmacological treatments for PTSD, the SSRIs, act through multiple molecular mechanisms other than by inhibiting serotonin reuptake. These mechanisms include the stimulation of neurosteroid and endocannabinoid biosynthesis and neurotrophic factors, such as BDNF, which are found deficient in PTSD. Increasing allopregnanolone levels is also associated with increased BDNF expression (Nin et al., 2011a). Collectively, these findings have contributed to improve our understanding of the psychobiological abnormalities associated with PTSD and promote the development of novel targeted treatment options. For instance, the correlation between the impairment of neurosteroid biosynthesis and behavioral modifications in neuropsychiatric disorders has been the focus of several studies (van Broekhoven and Verkes, 2003; reviewed in Pinna, 2013; Agis-Balboa et al., 2014; Locci and Pinna, 2017a). A reduction in the content of the GABAergic modulator allopregnanolone and its equipotent isomer pregnanolone was reported in cerebrospinal fluid (CSF) and serum of major depression and PTSD patients (Romeo et al., 1998; Uzunova et al., 1998; Rasmusson et al., 2006, 2016; Pineles et al., 2018). A negative correlation between CSF allopregnanolone levels and PTSD symptoms was more recently confirmed in male patients (Rasmusson et al., 2018). Other clinical studies support the significance of allopregnanolone biosynthesis as a biomarker of mood disorders (Uzunova et al., 1998; Agis-Balboa et al., 2014; reviewed in Zorumski et al., 2013; Schüle et al., 2014; Locci and Pinna, 2017a) with finding showing decreased allopregnanolone levels in postpartum depression (Nemeroff, 2008), under treatment with finasteride, an allopregnanolone biosynthetic enzyme blocker (Altomare and Capella, 2002; Caruso, 2015; Welk et al., 2017), and with anorexia nervosa or obesity complicated by anxiety and depression (Dichtel et al., 2018). Intriguingly, SSRI treatments normalize plasma, CSF, and brain allopregnanolone content in association with improvement of symptoms in responders only (Romeo et al., 1998; Uzunova et al., 1998; Agis-Balboa et al., 2014). These findings are in support of the role of allopregnanolone in the mechanisms of SSRIs' anxiolytic effects (Pinna, 2015). The downregulation of neurosteroid levels found in PTSD and depressed patients can be modeled in rodents exposed to protracted stress, including the socially-isolated mouse. Allopregnanolone is produced in brain corticolimbic neurons (Figure 1) and a reduction of its levels by prolonged social isolation (Agís-Balboa et al., 2006, 2007) or exposure to single prolonged stressors, results in development of anxiety-like behavior, aggression and enhanced contextual fear conditioning responses associated with impairment of fear extinction and elevated spontaneous fear responses at recall (Pinna et al., 2003; Pibiri et al., 2008; Pinna and Rasmusson, 2014; Qiu et al., 2015). These preclinical studies further support allopregnanolone as a putative *biomarker* for stress-induced emotional modification, such as exaggerated fear responses and impaired fear extinction, a hallmark in PTSD (Pibiri et al., 2008; Pinna et al., 2008; Pinna and Rasmusson, 2012). This evidence also suggests that new therapeutic approaches should counteract the downregulation of neurosteroid biosynthesis to improve symptoms in PTSD patients. In recent phase 3 clinical trials, intravenous allopregnanolone (brexanolone or SAGE-547) or an oral analog (SAGE-217) showed a rapid and long-lasting remission of post-partum depression and major depressive disorder symptoms, conditions highly comorbid with PTSD (Kanes et al., 2017; http://investor.sagerx.com/news-releases/news-release-details/sage-announces-pivotal-phase-3-trial-status-sage-217-major). If successfully developed, SAGE-217 will be the first durable, rapid-acting, oral, short-course treatment for mood disorders with potential application for PTSD treatment. Stress tremendously affects the expression of GABA_A_ receptor subunits (Pinna et al., 2006; reviewed in Locci and Pinna, 2017a). After social isolation, the α4, α5, and δ subunit expression was increased, and the α1, α2, and γ2 were significantly decreased in corticolimbic areas. These changes result in decreased benzodiazepine recognition sites and lower pharmacological response to benzodiazepines (Pinna et al., 2006). Remarkably, protracted stress favors a GABA_A_ receptor composition with high sensitivity for allopregnanolone and its analogs (Locci et al., 2017; reviewed in Locci and Pinna, 2017a). Clinical findings support lower benzodiazepine recognition site binding in brain of PTSD patients in association with benzodiazepine-insensitivity (Geuze et al., 2008). Altogether, these findings suggest that isolation stress results in: (i) changes in GABA_A_ receptor subunit composition; (ii) downregulated neurosteroidogenesis; and (iii) lack of response to benzodiazepines, which may provide a unique *biomarker axis* for PTSD (Figure 2). Allopregnanolone, analogs or stimulation of allopregnanolone biosynthesis may be a valuable therapeutic strategy for stress-induced psychiatric disorders, characterized by benzodiazepine-inefficacy and poor response to SSRIs. The pharmacological profile of SSRIs on stimulation of neurotropic factors, including the brain-derived neurotrophic factor (BDNF), via stimulation of allopregnanolone biosynthesis is an additional important mechanism to consider when establishing biomarkers for PTSD. BDNF expression decrease in PTSD patients is associated with symptom severity. In the socially isolated mouse, fluoxetine improves behavior by elevating the corticolimbic levels of allopregnanolone and BDNF expression, independently from the action of these drugs on serotonin reuptake inhibition. This and other evidence suggest that neurosteroid biosynthesis and BDNF expression may be interrelated (Nin et al., 2011a; Frye et al., 2014), and this may provide further support for biomarker selection.

Hence, biomarkers that instruct which treatment would be most effective for a patient is expected to considerably reduce non-responders and non-completers rate. Discovering new targets and agents to stimulate allopregnanolone biosynthesis is pivotal in this process. While more research is required to elucidate the interaction between the endocannabinoid system and allopregnanolone biosynthesis and specifically, following activation of PPAR-α, undoubtedly their cross-talk offers a unique opportunity to assess a biomarker axis that encompasses these two systems (Figure 2). A better assessment can be done following clarification of how stress affects PPAR-α expression and function. The concentrations of its main endogenous modulators, PEA, OEA, and SEA, which were found decreased in hair of PTSD patients, should be confirmed systemically in serum and CSF of patients. Preclinical studies should also verify whether their biosynthesis is altered in brain of rodent models of stress-induced behavioral deficits. Furthermore, methods development to simultaneously determine both endocannabinoid (AEA), cannabinoid-like (PEA) and neurosteroid (allopregnanolone) concentrations in same samples will consistently enhance the understanding on how the two systems are coordinated in neuropsychiatric disorders.

Both endocannabinoids and neurosteroids can be measured by GC-MS, however, presently there is no method that can determine them simultaneously in the same samples. Our laboratory established GC-MS methods to determine neurosteroids and their sulfates in human and rodent samples and we are currently developing new methods to include the quantification of endocannabinoids with demonstrated involvement in the pathophysiology of PTSD. Hence, these studies will clarify whether allopregnanolone levels down-regulation is causally linked to a PPAR-α expression down-regulation and/or endocannabinoid concentrations. The goal is to provide a reliable bio-signature that may uniquely define neurobiological alteration in PTSD and shows diagnostic validity.

While many aspects that relate to the endocannabinoid and neurosteroid cross-talk remain presently obscure, our current findings highlight the potential for: (i) assessing novel biomarkers to predict, diagnose, and treat PTSD at the interface of the PPAR-α-allopregnanolone axis; and (ii) repurposing FDA-approved PPAR-α agonists for the treatment of PTSD after positive clinical trials. Very few drugs are direct agonists of PPAR-α, and none have been tested for their potential effects in fear responses. However, one class of drugs, the fibrates are fibric acid derivatives that are prescribed to lower plasma lipids and triglyceride levels and are synthetic PPAR-α agonists that may be exploited in rodent models of PTSD to improve behavioral deficits.

## Conclusions

Progress in assessing biomarkers to predict PTSD and its treatment response will guide the future of novel PTSD medications that may be designed to improve neurotransmission (GABA, NMDA), and neuroendocrinologic (allopregnanolone biosynthesis) and anti-inflammatory (PPAR-α) responses. Research supports precision medicine for PTSD designed to stimulate neurosteroidogenesis after assessing in subpopulations of PTSD patients a downregulation of allopregnanolone biosynthesis. This can be achieved by acting at neurosteroidogenic targets or by mimicking allopregnanolone's function (e.g., analogs). Several neuronal targets to enhance steroidogenesis have recently been discovered and these include the endocannabinoid target PPAR-α. The crosstalk between the endocannabinoid system and the biosynthesis of neurosteroids, involving their targeted receptors or the biosynthetic enzymes promises to provide unique bio-signatures for stress-induced disorders.

Collectively, advances in the field suggest biomarker-based diagnosis and treatments for PTSD that encompass the neurosteroid and endocannabinoid systems may not be a far reach and these may provide a pivotal complement to the current practice of assessing the disorder based on self-reported symptoms and psychiatrist assessments.

## Author contributions

The author confirms being the sole contributor of this work and approved it for publication.

### Conflict of interest statement

The author declares that the research was conducted in the absence of any commercial or financial relationships that could be construed as a potential conflict of interest.

## References

[B1] Adamczyk P. Gołda A. McCreary A. C. Filip M. Przegalinski E. (2008). Activation of endocannabinoid transmission induces antidepressant-like effects in rats. J. Physiol. Pharmacol. 59, 217–228. 18622041

[B2] Agis-Balboa R. C. Guidotti A. Pinna G. (2014). Allopregnanolone biosynthesis is downregulated in the prefrontal cortex/Brodmann's area 9 (BA9) of depressed patients. Psychopharmacology 231, 3569–3580. 10.1007/s00213-014-3567-5 24781515 PMC6223254

[B3] Agís-Balboa R. C. Pinna G. Zhubi A. Maloku E. Veldic M. Costa E. (2006). Characterization of brain neurons that express enzymes mediating neurosteroid biosynthesis. Proc. Natl. Acad. Sci. U.S.A. 103, 14602–14607. 10.1073/pnas.0606544103 16984997 PMC1600006

[B4] Agís-Balboa R. C. Pinna G. Pibiri F. Kadriu B. Costa E. Guidotti A. (2007). Down-regulation of neurosteroid biosynthesis in corticolimbic circuits mediates social isolation-induced behavior in mice. Proc. Natl. Acad. Sci. U.S.A. 104, 18736–18741. 10.1073/pnas.0709419104 18003893 PMC2141846

[B5] Altomare G. Capella G. L. (2002). Depression circumstantially related to the administration of finasteride for androgenetic alopecia. J. Dermatol. 29, 665–669. 10.1111/j.1346-8138.2002.tb00200.x 12433001

[B6] Belelli D. Lambert J. J. (2005). Neurosteroids: endogenous regulators of the GABA(A) receptor. Nat. Rev. Neurosci. 6, 565–575. 10.1038/nrn1703 15959466

[B7] Bernardy N. C. Friedman M. J. (2017). Pharmacological management of posttraumatic stress disorders. Curr. Opin. Psychol. 14, 116–121. 10.1016/j.copsyc.2017.01.003 28813308

[B8] Borovska J. Vyklicky V. Stastna E. Kapras V. Slavikova B. Horak M.. (2012). Access of inhibitory neurosteroids to the NMDA receptor. Br. J. Pharmacol. 166, 1069–1083. 10.1111/j.1476-5381.2011.01816.x 22188257 PMC3417430

[B9] Brewin C. R. Cloitre M. Hyland P. Shevlin M. Maercker A. Bryant R. A. (2017). A review of current evidence regarding the ICD-11 proposals for diagnosing PTSD and complex PTSD. Clin. Psychol. Rev. 58, 1–15. 10.1016/j.cpr.2017.09.001 29029837

[B10] Brown N. Kerby J. Bonnert T. P. Whiting P. J. Wafford K. A. (2002). Pharmacological characterization of a novel cell line expressing human alpha(4)beta(3)delta GABA(A) receptors. Br. J. Pharmacol. 136, 965–974. 10.1038/sj.bjp.0704795 12145096 PMC1573424

[B11] Caruso D. (2015). Patients treated for male pattern hair with finasteride show, after discontinuation of the drug, altered levels of neuroactive steroids in cerebrospinal fluid and plasma. J. Steroid Biochem. Mol. Biol. 146, 74–79. 10.1016/j.jsbmb.2014.03.012 24717976

[B12] Chhatwal J. P. Davis M. Maguschak K. A. Ressler K. J. (2005). Enhancing cannabinoid neurotransmission augments the extinction of conditioned fear. Neuropsychopharmacology 30, 516–524. 10.1038/sj.npp.1300655 15637635

[B13] D'Agostino G. (2007). Acute intracerebroventricular administration of palmitoylethanolamide, an endogenous peroxisome proliferator-activated receptor-alpha agonist, modulates carrageenan-induced paw edema in mice. J. Pharmacol. Exp. Ther. 322, 1137–1143. 10.1124/jpet.107.123265 17565008

[B14] D'Agostino G. La Rana G. Russo R. Sasso O. Iacono A. Esposito E.. (2009). Central administration of palmitoylethanolamide reduces hyperalgesia in mice via inhibition of NF-κB nuclear signalling in dorsal root ganglia. Eur. J. Pharmacol. 613, 54–59. 10.1016/j.ejphar.2009.04.022 19386271

[B15] Dichtel L. E. Lawson E. A. Schorr M. Meenaghan E. Paskal M. L. Eddy K. T.. (2018). Neuroactive steroids and affective symptoms in women across the weight spectrum. Neuropsychopharmacology. 10.1038/npp.2017.269 29090684 PMC5916351

[B16] Dlugos A. (2012). Acute stress increases circulating anandamide and other N-acylethanolamines in healthy humans. Neuropsychopharmacology 37, 2416–2427. 10.1038/npp.2012.100 22763622 PMC3442338

[B17] Dubreucq S. Matias I. Cardinal P. Häring M. Lutz B. Marsicano G.. (2012). Genetic dissection of the role of cannabinoid type-1 receptors in the emotional consequences of repeated social stress in mice. Neuropsychopharmacology 37, 1885–1900. 10.1038/npp.2012.36 22434220 PMC3376321

[B18] Fernandes B. S. Williams L. M. Steiner J. Leboyer M. Carvalho A. F. Berk M. (2017). The new field of 'precision psychiatry'. BMC Med. 15:80. 10.1186/s12916-017-0849-x 28403846 PMC5390384

[B19] Franklin C. L. Raines A. M. Cuccurullo L. J. Chambliss J. L. Maieritsch K. P. Tompkins A. M. (2017). 27 ways to meet PTSD: using the PTSD-checklist for DSM-5 to examine PTSD core criteria. Psychiatry Res. 261, 504–507. 10.1016/j.psychres.2018.01.021 29395872

[B20] Frye C. A. Koonce C. J. Walf A. A. (2014). Involvement of pregnane xenobiotic receptor in mating-induced allopregnanolone formation in the midbrain and hippocampus and brain-derived neurotrophic factor in the hippocampus among female rats. Psychopharmacology (Berl). 231, 3375–3390. 10.1007/s00213-014-3569-3 24781516 PMC4135012

[B21] Geuze E. van Berckel B. N. Lammertsma A. A. Boellaard R. de Kloet C. S. Vermetten E. (2008). Reduced GABA-A benzodiazepine receptor binding in veterans with post-traumatic stress disorder. Mol. Psychiatry 13, 74–83. 10.1038/sj.mp.4002054 17667960

[B22] Ghazizadeh-Hashemi M. (2018). Palmitoylethanolamide as adjunctive therapy in major depressive disorder: a double-blind, randomized and placebo-controlled trial. J. Affect. Disord. 232, 127–133. 10.1016/j.jad.2018.02.057 29486338

[B23] Golden R. N. Nemeroff C. B. McSorley P. Pitts C. D. Dubé E. M. (2002). Efficacy and tolerability of controlled-release and immediate-release paroxetine in the treatment of depression. J. Clin. Psychiatry 63, 577–584. 10.4088/JCP.v63n0707 12143913

[B24] Guidotti A. Dong E. Matsumoto K. Pinna G. Rasmusson A. M. Costa E. (2001). The socially-isolated mouse: a model to study the putative role of allopregnanolone and 5alpha-dihydroprogesterone in psychiatric disorders. Brain Res. Brain Res. Rev. 37, 110–115. 10.1016/S0165-0173(01)00129-1 11744079

[B25] Häring M. Guggenhuber S. Lutz B. (2012). Neuronal populations mediating the effects of endocannabinoids on stress and emotionality. Neuroscience 1, 145–158. 10.1016/j.neuroscience.2011.12.035 22233782

[B26] Heyman E. (2012). Intense exercise increases circulating endocannabinoid and BDNF levels in humans-possible implications for reward and depression. Psychoneuroendocrinology 37, 844–851. 10.1016/j.psyneuen.2011.09.017 22029953

[B27] Hill M. N. Hillard C. J. Bambico F. R. Patel S. Gorzalka B. B. Gobbi G. (2009b). The therapeutic potential of the endocannabinoid system for the development of a novel class of antidepressants. Trends Pharmacol. Sci. 30, 484–493. 10.1016/j.tips.2009.06.006 19732971

[B28] Hill M. N. Miller G. E. Carrier E. J. Gorzalka B. B. Hillard C. J. (2009a). Circulating endocannabinoids and N-acyl ethanolamines are differentially regulated in major depression and following exposure to social stress. Psychoneuroendocrinology 34, 1257–1262. 10.1016/j.psyneuen.2009.03.013 19394765 PMC2716432

[B29] Hill M. N. Patel S. (2013). Translational evidence for the involvement of the endocannabinoid system in stress-related psychiatric illnesses. Biol. Mood Anxiety Disord. 3:19. 10.1186/2045-5380-3-19 24286185 PMC3817535

[B30] Holman E. A. Guijarro A. Lim J. Piomelli D. (2014). Effects of acute stress on cardiac endocannabinoids, lipogenesis, and inflammation in rats. Psychosom. Med. 76, 20–28. 10.1097/PSY.0000000000000025 24367128 PMC3988664

[B31] Hosie A. M. Wilkins M. E. da Silva H. M. Smart T. G. (2006). Endogenous neurosteroids regulate GABAA receptors through two discrete transmembrane sites. Nature 444, 486–489. 10.1038/nature05324 17108970

[B32] Jacob W. Marsch R. Marsicano G. Lutz B. Wotjak C. T. (2012). Cannabinoid CB1 receptor deficiency increases contextual fear memory under highly aversive conditions and long-term potentiation *in vivo*. Neurobiol. Learn. Mem. 98, 47–55. 10.1016/j.nlm.2012.04.008 22579951

[B33] Kamprath K. Marsicano G. Tang J. Monory K. Bisogno T. Di Marzo V.. (2006). Cannabinoid CB1 receptor mediates fear extinction via habituation-like processes. J. Neurosci. 26, 6677–6686. 10.1523/JNEUROSCI.0153-06.2006 16793875 PMC6673838

[B34] Kanes S. Colquhoun H. Gunduz-Bruce H. Raines S. Arnold R. Schacterle A. (2017). Brexanolone (SAGE-547 injection) in post-partum depression: a randomized controlled trial. Lancet 390, 480–489. 10.1016/S0140-6736(17)31264-3 28619476

[B35] Katona I. (2009) Endocannabinoid receptors: CNS localization of the CB1 cannabinoid receptor. Curr. Top Behav. Neurosci. 1, 65–86. 10.1007/978-3-540-88955-7_3 21104380

[B36] Kemp A. H. Gordon E. Rush A. J. Williams L. M. (2008). Improving the prediction of treatment response in depression: integration of clinical, cognitive, psychophysiological, neuroimaging, and genetic measures. CNS Spectr. 13, 1066–1086. 10.1017/S1092852900017120 19179943

[B37] Lo Verme J. Fu J. Astarita G. La Rana G. Russo R. Calignano A.. (2005). The nuclear receptor peroxisome proliferator-activated receptor-alpha mediates the anti-inflammatory actions of palmitoylethanolamide. Mol. Pharmacol. 67, 15–19. 10.1124/mol.104.006353 15465922

[B38] Locci A. Pinna G. (2017a). Neurosteroid biosynthesis down-regulation and changes in GABAA receptor subunit composition: a biomarker axis in stress-induced cognitive and emotional impairment. Br. J. Pharmacol. 174, 3226–3241. 10.1111/bph.13843 28456011 PMC5595768

[B39] Locci A. Pinna G. (2017b). Stimulation of the endocannabinoid system by PEA engages neurosteroid biosynthesis to improve anxiety and fear in a PTSD mouse model. 165.19, in 47th Annual Meeting Society for Neuroscience (Washington, DC).

[B40] Locci A. Geoffroy P. Miesch M. Mensah-Nyagan A.-G. Pinna G. (2017). Social isolation in early versus late adolescent mice is associated with persistent behavioral deficits that can be improved by neurosteroid-based treatment. Front. Cell Neurosci. 11:208. 10.3389/fncel.2017.00208 28900387 PMC5581875

[B41] Locci A. Khan F. Khan M. A. Pinna G. (2018). Neurosteroid-based biomarkers and therapeutic approaches to facilitate resilience after trauma, in Facilitating Fear After Trauma: A Translational Approach, eds Pinna G. Izumi T. (New York, NY: Nova Biomedical Publication).

[B42] Lovick T. (2013). SSRIs and the female brain–potential for utilizing steroid-stimulating properties to treat menstrual cycle-linked dysphorias. J. Psychopharmacol. 27, 1180–1185. 10.1177/0269881113490327 23704364

[B43] Malayev A. Gibbs T. T. Farb D. H. (2002). Inhibition of the NMDA response by pregnenolone sulphate reveals subtype selective modulation of NMDA receptors by sulphated steroids. Br. J. Pharmacol. 135, 901–909. 10.1038/sj.bjp.0704543 11861317 PMC1573207

[B44] Marsicano G. Wotjak C. T. Azad S. C. Bisogno T. Rammes G. Cascio M. G.. (2002). The endogenous cannabinoid system controls extinction of aversive memories. Nature 418, 530–534. 10.1038/nature00839 12152079

[B45] Melis M. Carta G. Pistis M. Banni S. (2013). Physiological role of peroxisome proliferator-activated receptors type alpha on dopamine systems. CNS Neurol Disord Drug Targets. 12, 70–77. 10.2174/1871527311312010012 23394525

[B46] Moreno S. Farioli-Vecchioli S. Cerù M. P. (2004). Immunolocalization of peroxisome proliferator-activated receptors and retinoid X receptors in the adult rat CNS. Neuroscience 123, 131–145. 10.1016/j.neuroscience.2003.08.064 14667448

[B47] Nemeroff C. B. (2008). Understanding the pathophysiology of postpartum depression: implications for the development of novel treatments. Neuron 59, 185–186. 10.1016/j.neuron.2008.07.015 18667144

[B48] Neumeister A. (2013). The endocannabinoid system provides an avenue for evidence-based treatment development for PTSD Depress Anxiety. 30, 93–96. 10.1002/da.22031 23225490

[B49] Neumeister A. Seidel J. Ragen B. J. Pietrzak R. H. (2014). Translational evidence for a role of endocannabinoids in the etiology and treatment of posttraumatic stress disorder. Psychoneuroendocrinology 51, 577–584. 10.1016/j.psyneuen.2014.10.012 25456347 PMC4268027

[B50] Ngounou Wetie A. G. Sokolowska I. Wormwood K. Beglinger K. Michel T. M. Thome J.. (2013). Mass spectrometry for the detection of potential psychiatric biomarkers. J. Mol. Psychiatry 1:8. 10.1186/2049-9256-1-8 25408901 PMC4223884

[B51] Nin M. S. Martinez L. A. Thomas R. Nelson M. Pinna G. (2011b). Allopregnanolone and S-norfluoxetine decrease anxiety-like behavior in a mouse model of anxiety/depression. Trabajos del Instituto Cajal 83, 215–216.

[B52] Nin M. S. Martinez L. A. Pibiri F. Nelson M. Pinna G. (2011a). Neurosteroids reduce social isolation-induced behavioral deficits: a proposed link with neurosteroid-mediated upregulation of BDNF expression. Front. Endocrin. 2:73. 10.3389/fendo.2011.00073 22649384 PMC3355888

[B53] Nusser Z. Mody I. (2002). Selective modulation of tonic and phasic inhibitions in dentate gyrus granule cells. J. Neurophysiol. 87, 2624–2628. 10.1152/jn.2002.87.5.2624 11976398

[B54] O'Sullivan S. E. (2007). Cannabinoids go nuclear: evidence for activation of peroxisome proliferator-activated receptors. Br. J. Pharmacol. 152, 576–582. 10.1038/sj.bjp.0707423 17704824 PMC2190029

[B55] Papini S. Sullivan G. M. Hien D. A. Shvil E. Neria Y. (2015). Toward a translational approach to targeting the endocannabinoid system in posttraumatic stress disorder: a critical review of preclinical research. Biol. Psychol. 104, 8–18. 10.1016/j.biopsycho.2014.10.010 25448242 PMC4465924

[B56] Park-Chung M. Malayev A. Purdy R. H. Gibbs T. T. Farb D. H. (1999). Sulfated and unsulfated steroids modulate gamma-aminobutyric acidA receptor function through distinct sites. Brain Res. 830, 72–87. 10.1016/S0006-8993(99)01381-5 10350561

[B57] Patel S. Carrier E. J. Ho W. S. Rademacher D. J. Cunningham S. Reddy D. S.. (2005). The postmortal accumulation of brain N-arachidonylethanolamine (anandamide) is dependent upon fatty acid amide hydrolase activity. J. Lipid Res. 46, 342–349. 10.1194/jlr.M400377-JLR200 15576840

[B58] Petrosino S. Di Marzo V. (2017). The pharmacology of palmitoylethanolamide and first data on the therapeutic efficacy of some of its new formulations. Br. J. Pharmacol. 174, 1349–1365. 10.1111/bph.13580 27539936 PMC5429331

[B59] Pibiri F. Nelson M. Guidotti A. Costa E. Pinna G. (2008). Decreased corticolimbic allopregnanolone expression during social isolation enhances contextual fear: a model relevant for posttraumatic stress disorder. Proc. Natl. Acad. Sci. U.S.A. 105, 5567–5572. 10.1073/pnas.0801853105 18391192 PMC2291140

[B60] Pineles S. L. Nillni Y. I. Pinna G. Irvine J. Webb A. Hall A. (2018). PTSD in women is associated with a block in conversion of progesterone to the GABAergic neurosteroids allopregnanolone and pregnanolone: confirmed in plasma. Psychoneuroendocrinology 93, 133–141. 10.1016/j.psyneuen.2018.04.024 29727810

[B61] Pinna G. (2013). Targeting neurosteroidogenesis as therapy for PTSD. *Front*. Pharmacol. 4:166 10.3389/fphar.2013.00166 PMC3880842 24432002

[B62] Pinna G. (2015). The Neurosteroidogenic Action of Fluoxetine Unveils the Mechanism for the Anxiolytic Property of SSRIs, in Fluoxetine: Pharmacology, Mechanisms of Action and Potential Side Effects, Pharmacology - Research, Safety Testing and Regulation Series, ed Pinna G. (New York, NY: Nova Biomedical Publication).

[B63] Pinna G. Agis-Balboa R. C. Pibiri F. Nelson M. Guidotti A. Costa E. (2008). Neurosteroid biosynthesis regulates sexually dimorphic fear and aggressive behavior in mice. Neurochem. Res. 33, 1990–2007. 10.1007/s11064-008-9718-5 18473173

[B64] Pinna G. Agis-Balboa R. C. Zhubi A. Matsumoto K. Grayson D. R. Costa E.. (2006). Imidazenil and diazepam increase locomotor activity in mice exposed to protracted social isolation. Proc. Natl. Acad. Sci. U.S.A. 103, 4275–4280. 10.1073/pnas.0600329103 16537521 PMC1449683

[B65] Pinna G. Izumi T. (2018). Biomarkers for resilience after trauma: a translational approach, in Preface Facilitating Fear After Trauma: A Translational Approach, eds Pinna G. Izumi T. (New York, NY: Nova Biomedical Publication).

[B66] Pinna G. Rasmusson A. (2014). Ganaxolone improves behavioral deficits in a mouse model of post-traumatic stress disorder. Front. Cell. Neurosci. 8:256. 10.3389/fncel.2014.00256 25309317 PMC4161165

[B67] Pinna G. Rasmusson A. M. (2012). Upregulation of neurosteroid biosynthesis as a pharmacological strategy to improve behavioral deficits in a putative mouse model of PTSD. J. Neuroendocrinol. 24, 102–116. 10.1111/j.1365-2826.2011.02234.x 21981145 PMC3245370

[B68] Pinna G. Dong E. Matsumoto K. Costa E. Guidotti A. (2003). In socially isolated mice, the reversal of brain allopregnanolone down-regulation mediates the anti-aggressive action of fluoxetine. Proc. Natl. Acad. Sci. U.S.A. 100, 2035–2040. 10.1073/pnas.0337642100 12571361 PMC149954

[B69] Pinna G. Uzunova V. Matsumoto K. Puia G. Mienville J. M. Costa E.. (2000). Brain allopregnanolone regulates the potency of the GABA(A) receptor agonist muscimol. Neuropharmacology 39, 440–448. 10.1016/S0028-3908(99)00149-5 10698010

[B70] Pistis M. Melis M. (2010). From surface to nuclear receptors: the endocannabinoid family extends its assets. Curr. Med. Chem. 17, 1450–1467. 10.2174/092986710790980014 20166922

[B71] Qiu Z. K. Zhang G. H. He J. L. Ma J. C. Zeng J. Shen D.. (2015). Free and Easy Wanderer Plus (FEWP) improves behavioral deficits in an animal model of post-traumatic stress disorder by stimulating allopregnanolone biosynthesis. Neurosci. Lett. 602, 162–166. 10.1016/j.neulet.2015.06.055 26160034

[B72] Rasmusson A. M. King M. Gregor K. Scioli-Salter E. Pineles S. Valovski I. (2016). Sex differences in the enzyme site at which GABAergic neuroactive steroid synthesis is blocked in PTSD: implications for targeting of PTSD therapeutics, in Symposium: Sex Specificity in Posttraumatic Stress Disorder: From Biological Mechanisms to Treatment Response, eds Chair F. K. Discussant J. T. (Dallas, TX: 32nd Annual Meeting, International Society for Traumatic Stress Studies).

[B73] Rasmusson A. M. King M. Gregor K. Scioli-Salter E. Pineles S. Valovski I. (2018). GABAergic neurosteroids in cerebrospinal fluid are negatively associated with PTSD severity in men. Biol. Psychiatry 83, S15–S16. 10.1016/j.biopsych.2018.02.055

[B74] Rasmusson A. M. Marx C. E. Pineles S. L. Locci A. Scioli-Salter E. R. Nillni Y. I.. (2017). Neuroactive steroids and PTSD treatment. Neurosci. Lett. 649, 156–163. 10.1016/j.neulet.2017.01.054 28215878

[B75] Rasmusson A. M. Pinna G. Paliwal P. Weisman D. Gottschalk C. Charney D.. (2006). Decreased cerebrospinal fluid allopregnanolone levels in women with posttraumatic stress disorder. Biol. Psychiatry 60, 704–713. 10.1016/j.biopsych.2006.03.026 16934764

[B76] Raso G. M. Esposito E. Vitiello S. Iacono A. Santoro A. D'Agostino G.. (2011). Palmitoylethanolamide stimulation induces allopregnanolone synthesis in C6 Cells and primary astrocytes: involvement of peroxisome-proliferator activated receptor-α. J. Neuroendocrinol. 23, 591–600. 10.1111/j.1365-2826.2011.02152.x 21554431

[B77] Reich C. G. Mohammadi M. H. Alger B. E. (2008). Endocannabinoid modulation of fear responses: learning and state-dependent performance effects. J. Psychopharmacol. 22, 769–777. 10.1177/0269881107083999 18308796 PMC2906780

[B78] Riebe C. J. Wotjak C. T. (2011). Endocannabinoids and stress. Stress 14, 384–397. 10.3109/10253890.2011.586753 21663537

[B79] Romeo E. Ströhle A. Spalletta G. di Michele F. Hermann B. Holsboer F.. (1998). Effects of antidepressant treatment on neuroactive steroids in major depression. Am. J. Psychiatry 155, 910–913. 10.1176/ajp.155.7.910 9659856

[B80] Ruehle S. Rey A. A. Remmer F. Lutz B. (2012). The endocannabinoid system in anxiety, fear memory and habituation. J. Psychopharmacol. 26, 23–39. 10.1177/0269881111408958 21768162 PMC3267552

[B81] Rush A. J. Bernstein I. H. Trivedi M. H. Carmody T. J. Wisniewski S. Mundt J. C.. (2006). An evaluation of the quick inventory of depressive symptomatology and the hamilton rating scale for depression: a sequenced treatment alternatives to relieve depression trial report. Biol. Psychiatry 59, 493–501. 10.1016/j.biopsych.2005.08.022 16199008 PMC2929841

[B82] Sasso O. La Rana G. Vitiello S. Russo R. D'Agostino G. Iacono A.. (2010). Palmitoylethanolamide modulates pentobarbital-evoked hypnotic effect in mice: involvement of allopregnanolone biosynthesis. Eur. Neuropsychopharmacol. 20, 195–206. 10.1016/j.euroneuro.2009.09.003 19864116

[B83] Sasso O. Russo R. Vitiello S. Raso G. M. D'Agostino G. Iacono A. (2012). Implication of allopregnanolone in the antinociceptive effect of N-palmitoylethanolamide in acute or persistent pain. Pain 153, 33–41. 10.1016/j.pain.2011.08.010 21890273

[B84] Schüle C. Nothdurfter C. Rupprecht R. (2014). The role of allopregnanolone in depression and anxiety. Prog. Neurobiol. 113, 79–87. 10.1016/j.pneurobio.2013.09.003 24215796

[B85] Shin L. M. Rauch S. L. Pitman R. K. (2006). Amygdala, medial prefrontal cortex, and hippocampal function in PTSD. Ann. N.Y. Acad. Sci. 1071, 67–79. 10.1196/annals.1364.007 16891563

[B86] Shu H. J. Bracamontes J. Taylor A. Wu K. Eaton M. M. Akk G.. (2012). Characteristics of concatemeric GABA(A) receptors containing α4/δ subunits expressed in Xenopus oocytes. Br. J. Pharmacol. 165, 2228–2243. 10.1111/j.1476-5381.2011.01690.x 21950777 PMC3413859

[B87] Smith C. C. Gibbs T. T. Farb D. H. (2014). Pregnenolone sulfate as a modulator of synaptic plasticity. Psychopharmacology (Berl) 231, 3537–3556. 10.1007/s00213-014-3643-x 24997854 PMC4625978

[B88] Stell B. M. Brickley S. G. Tang C. Y. Farrant M. Mody I. (2003). Neuroactive steroids reduce neuronal excitability by selectively enhancing tonic inhibition mediated by delta subunit-containing GABAA receptors. Proc. Natl. Acad. Sci. U.S.A. 100, 14439–14444. 10.1073/pnas.2435457100 14623958 PMC283610

[B89] Thiemann G. van der Stelt M. Petrosino S. Molleman A. Di Marzo V. Hasenöhrl R. U. (2008). The role of the CB1 cannabinoid receptor and its endogenous ligands, anandamide and 2-arachidonoylglycerol, in amphetamine-induced behavioural sensitization. Behav. Brain Res. 187, 289–296. 10.1016/j.bbr.2007.09.022 17988751

[B90] Trivisano M. Lucchi C. Rustichelli C. Terracciano A. Cusmai R. Ubertini G. M.. (2017). Reduced steroidogenesis in patients with PCDH19-female limited epilepsy. Epilepsia 58, e91–e95. 10.1111/epi.13772 28471529

[B91] Umathe S. N. Manna S. S. Jain N. S. (2011). Involvment of endocannabinoids in antidepressant and anti-compulsive effect of fluoxetine in mice. Behav. Brain. Res. 223, 125–134. 10.1016/j.bbr.2011.04.031 21549765

[B92] Uzunov D. P. Cooper T. B. Costa E. Guidotti A. (1996). Fluoxetine-elicited changes in brain neurosteroid content measured by negative ion mass fragmentography. Proc. Natl. Acad. Sci. U.S.A. 93, 12599–12604. 10.1073/pnas.93.22.12599 8901628 PMC38038

[B93] Uzunova V. Sheline Y. Davis J. M. Rasmusson A. Uzunov D. P. Costa E. (1998). Increase in the cerebrospinal fluid content of neurosteroids in patients with unipolar major depression who are receiving fluoxetine or fluvoxamine. Proc. Natl. Acad. Sci. U.S.A. 95, 3239–3244. 10.1073/pnas.95.6.3239 9501247 PMC19726

[B94] van Broekhoven F. Verkes R. J. (2003). Neurosteroids in depression: a review. Psychopharmacology 165, 97–110. 10.1007/s00213-002-1257-1 12420152

[B95] Viveros M. P. Marco E. M. File S. E. (2005). Endocannabinoid system and stress and anxiety responses. Pharmacol. Biochem. Behav. 81, 331–342. 10.1016/j.pbb.2005.01.029 15927244

[B96] Vyklicky V. Smejkalova T. Krausova B. Balik A. Korinek M. Borovska J.. (2016). Preferential Inhibition of tonically over phasically activated NMDA receptors by pregnane derivatives. J. Neurosci. 36, 2161–2175. 10.1523/JNEUROSCI.3181-15.2016 26888927 PMC6602038

[B97] Welk B. McArthur E. Ordon M. Anderson K. K. Hayward J. Dixon S. (2017). Association of suicidality and expression with 5α-reductase inhibitors. JAMA Int. Med. 177, 683–691. 10.1001/jamainternmed.2017.0089 PMC5818776 28319231

[B98] Wilker S. Pfeiffer A. Elbert T. Ovuga E. Karabatsiakis A. Krumbholz A.. (2016). Endocannabinoid concentrations in hair are associated with PTSD symptom severity. Psychoneuroendocrinology 67, 198–206. 10.1016/j.psyneuen.2016.02.010 26923850

[B99] Wohlfarth K. M. Bianchi M. T. Macdonald R. L. (2002). Enhanced neurosteroid potentiation of ternary GABA(A) receptors containing the delta subunit. J. Neurosci. 22, 1541–1549. 10.1523/JNEUROSCI.22-05-01541.2002 11880484 PMC6758857

[B100] Zorumski C. F. Paul S. M. Izumi Y. Covey D. F. Mennerick S. (2013). Neurosteroids, stress and depression: potential therapeutic opportunities. Neurosci. Biobehav. Rev. 37, 109–122. 10.1016/j.neubiorev.2012.10.005 23085210 PMC3591791

